# Effects of personalized time-trial goal setting on swimming performance and psychological profiles in competitive swimmers

**DOI:** 10.3389/fspor.2026.1759066

**Published:** 2026-03-11

**Authors:** Nadhir Ferchichi, Sofiene Amara, Hajer Aouani, Anissa Bouassida, Roland van den Tillaar

**Affiliations:** 1 Higher Institute of Sport and Physical Education, University of Jendouba, Kef, Tunisia; 2 Research Laboratory Sports Performance Optimization (LR09SEP01), National Center of Medicine and Science in Sports (CNMSS), Tunis, Tunisia; 3 Department of Sports Science and Physical Education, Nord University, Levanger, Norway

**Keywords:** competitive anxiety, interval of goal setting, mood disturbance, psycho-physiological, training intervention

## Abstract

Swimming performance results from a complex interaction between physiological and psychological factors. The use of specific goal-setting has emerged as a promising psychological strategy; however, its influence on emotional states and time-based performance in real competitive conditions remains insufficiently documented. The aim of this study was to investigate the effect of setting a specific time-based performance goal on 100-m freestyle performance and psychological states in competitive swimmers. Twenty-four male swimmers (15.2 ± 0.8 years) were randomly assigned to an experimental group (*n* = 12) or a control group (*n* = 12). The experimental group received an individualized performance goal based on the goal-range setting model, whereas the control group was instructed to “perform at their best.” All swimmers completed an 8-week standardized training program. Performance in the 100-m freestyle and psychological states (POMS) were assessed pre- and post-intervention. The mixed ANOVA revealed a significant main effect of time on performance (*p* < 0.001) and a significant Time × Group interaction (*p* = 0.004). Both groups improved following the intervention; however, the improvement was greater in the experimental group (4.3% vs. 2.5%). Regarding psychological variables, a significant main effect of time was found for total mood disturbance (*p* < 0.001), along with a significant Time × Group interaction (*p* < 0.001). Total mood disturbance increased in both groups, but to a greater extent in the experimental group (47.1% vs. 19.1%). All POMS subscales demonstrated significant effects of time and interaction, except for fatigue (*p* = 0.143). *Post hoc* analyses showed significantly greater increases in all affective states in the experimental group, while fatigue did not differ significantly between groups (13.7% vs. 9.3%). Implementing an individualized time-based performance goal was associated with improved swimming performance; however, it was also accompanied by a substantial emotional cost, reflecting an imbalance between performance demands and available psychological resources. The use of individualized goals should be coupled with structured psychological monitoring, including regular mood profiling, emotional regulation strategies, and progressive co-construction of the performance target. A gradual implementation approach appears necessary to optimize performance gains while preserving athlete well-being.

## 1 Introduction

Competitive swimming performance results from the interaction of physiological, biomechanical, and psychological determinants, making successful performance multidimensional and complex (1, 2). While traditional performance models have primarily emphasized physiological factors such as aerobic capacity, anaerobic power, and technical efficiency, growing evidence highlights the critical role of psychological processes in regulating performance, particularly under competitive pressure (3, 4). Given the time-constrained and precision-dependent nature of swimming events, psychological fluctuations may substantially influence motor execution, race strategy, and performance stability (5, 6). Accordingly, understanding and integrating psychological regulation strategies into training programs appears essential for optimizing competitive swimming performance.

Athletes’ psychological states vary markedly between training and competitive phases, with swimmers frequently reporting higher levels of anxiety, psychological tension, and emotional disturbance approaching competition (7, 8). In swimmers, excessive anxiety is associated with reduced motor coordination, impaired hydrodynamic efficiency, and disruptions in stroke rhythm, ultimately affecting performance stability (9). Conversely, confidence, emotional calmness, and focused attention significantly improve race strategy, attentional control, and movement economy (10, 11). Longitudinal findings indicate that swimmers may experience 35%–60% variation in emotional states between training and competition (12), reflecting considerable psychological instability across a competitive season. These findings highlight the necessity of systematically integrating psychological regulation strategies into training programs, thereby justifying the investigation of targeted interventions in this area.

Mental preparation, including motor imagery, self-talk, arousal regulation, and goal setting, is widely recognized as an effective method for improving emotional regulation, motivation, and performance in athletes (13, 14). Among these techniques, goal setting, and particularly the use of performance-based and measurable targets, is one of the most extensively documented strategies. It enhances attentional resource allocation, perceived self-efficacy, and persistence in effort (15, 16). In swimming, where success is determined to the hundredth of a second, establishing a precise time-based goal may serve as a particularly powerful lever to guide motivation, engagement, and emotional readiness (17, 18). Despite solid theoretical foundations, few studies have investigated the immediate effects of goal setting on psychological states and objective performance in real competitive settings. This gap reinforces the scientific need to examine the effect of goal setting on performance in swimming.

Based on evidence that sport performance is influenced by psychological states and that goal setting may function as an effective method of cognitive and motivational regulation, the present study aims to examine the effect of imposing a time-based performance goal on 100-m freestyle performance and associated psychological responses in competitive swimmers. More specifically, the study seeks to determine whether goal setting results in significant improvements in swim time, measurable variations in emotional states (tension, depression, anger, vigor, fatigue, and confusion), and a significant interaction between time and experimental condition. We hypothesize that swimmers exposed to goal setting will demonstrate (i) a significant improvement in performance, (ii) heightened psychological activation, and (iii) greater psychological fluctuations compared with the control group.

## 2 Materials and methods

### 2.1 Study design

**Figure 1:**
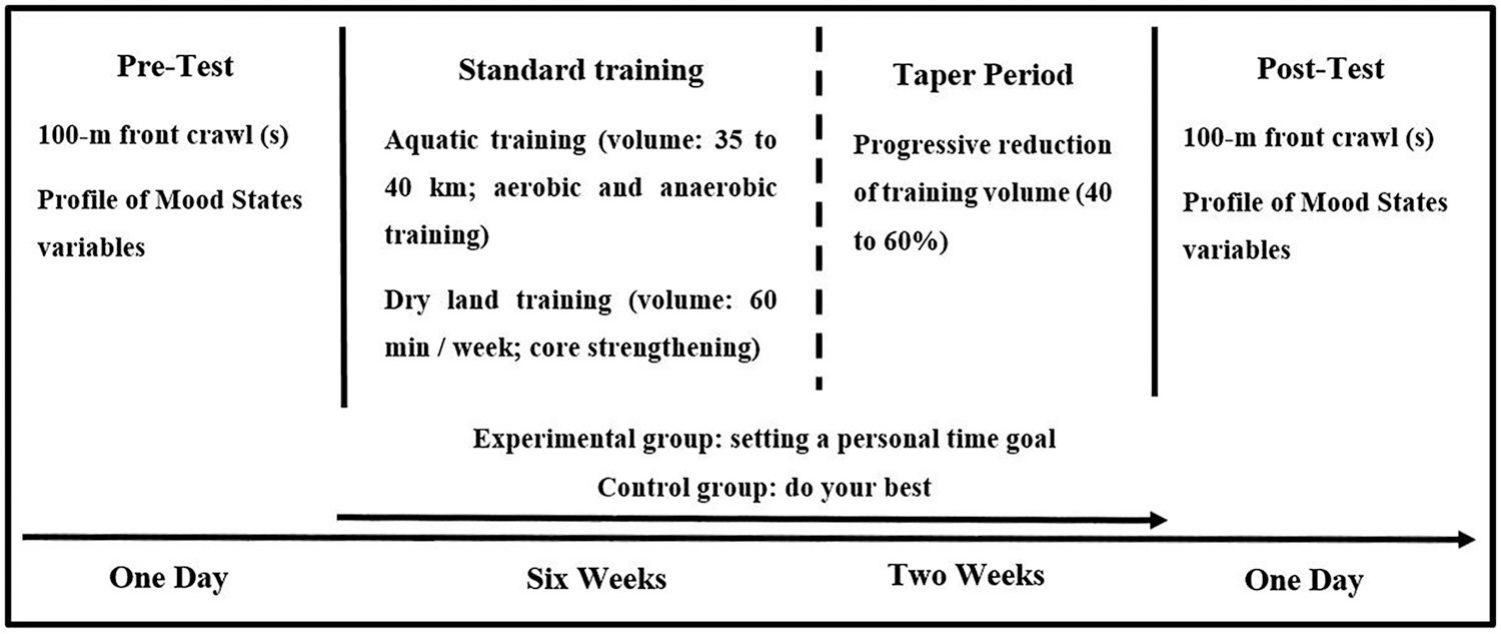
Schematic representation of the experimental study design and intervention timeline.

The present study employed an experimental design with a control group and pre–post testing. Immediately after the initial assessment (pre-test), which included a 100-m freestyle performance measurement and the administration of the POMS questionnaire, the experimental group received a personalized time-based performance goal to achieve during the post-test. The control group was instructed only to “do their best.” All participants then completed a standardized eight-week training period consisting of six weeks of regular training (including six in-water sessions and two dry-land sessions per week) followed by a two-week taper. Throughout the intervention period, swimmers in the experimental group received periodic reminders of their assigned performance goal. The post-test phase replicated the exact assessment protocol used in the pre-test (Figure 1).

### 2.2 Participants

Twenty-four competitive male swimmers (age: 15.2 ± 0.8 years; height: 1.75 ± 0.05 m; body mass: 64.8 ± 4.7 kg; BMI: 21.1 ± 1.0 kg/m^2^) volunteered to participate in this study. *A priori* power analysis conducted using G*Power (version 3.1.9.7) determined that a total sample size of *N* = 24 was required to detect a large interaction effect (f = 0.40) with a statistical power of 0.80 and *α* = 0.05 in a repeated-measures ANOVA design. Participants were randomly assigned to either the experimental group (*n* = 12, age: 15.1 ± 0.9 years, height: 1.76 ± 0.5 m, body mass: 65.2 ± 4.5 kg, BMI: 21.0 ± 1.1 kg/m^2^) or the control group (*n* = 12, age: 15.3 ± 0.7 years, height: 1.75 ± 0.05 m, body mass: 64.4 ± 5.0 kg, BMI: 21.1 ± 0.9 kg/m^2^). Inclusion criteria required swimmers to have a minimum of five years of competitive experience and to be ranked among the top 30 in national competition. Exclusion criteria included any injury affecting training or testing participation and the use of medication known to influence performance or mood. Written informed consent was obtained from all swimmers and their legal guardians prior to participation. The experimental protocol was approved by the institutional ethics committee of the Higher Institute of Sport and Physical Education of Kef, Tunisia (code ISSEPK: 24-2025) and adhered to the Declaration of Helsinki.

### 2.3 Standardized training

All participants followed the same standardized eight-week training program supervised by the same coaching staff. The program consisted of two distinct phases: six weeks of intensive maintenance training followed by a two-week taper period. During the intensive phase, swimmers completed eight weekly pool sessions, totaling approximately 35–40 km of training volume. External load was assessed through mean swimming distance and session duration, while internal load was quantified using session RPE and RPE load (Table 1). Sessions alternated between aerobic conditioning (long sets at 70%–80% of maximum heart rate), anaerobic lactic power development (50–100 m repetitions at maximal or supramaximal intensity), and technical drills, in accordance with established competitive swimming periodization principles (19, 20). Two 60-minute dry-land sessions per week were included, targeting core stabilization, power development using medicine balls, and light plyometric training to enhance functional force production without excessive muscular hypertrophy (21). During the two-week taper, total training volume was progressively and non-linearly reduced by 40%–60%, while intensity was maintained, a strategy shown to optimize performance gains while reducing fatigue (20, 22). This strict standardization ensured that any observed post-test differences could be attributed to the psychological intervention rather than discrepancies in training load.

**Table 1:** Descriptive statistics and mixed ANOVA outcomes for performance and psychological variables.

Variable	Groups	Pretest mean ± SD	Posttest mean ± SD	F	p	*η*^2^p	Effect group			Effect time			Effect time × group		
							F	*p*	η^2^p	F	*p*	η^2^p	F	*p*	η^2^p
100-m (s)	Exp	54.67 ± 2.43	52.30 ± 1.88	7.13	0.014	0.25	0.19	0.666	0.01	151.77	0.000	0.87	10.04	0.004	0.31
	Cont	54.60 ± 2.67	53.21 ± 2.52	1.74	0.201	0.07									
TMD	Exp	5.917 ± 1.38	8.50 ± 1.45	7.07	0.014	0.24	0.67	0.420	0.03	241.34	0.000	0.92	42.63	0.000	0.66
	Cont	6.08 ± 1.44	6.58 ± 1.16	1.59	0.221	0.07									
Tension	Exp	4.75 ± 1.36	6.92 ± 1.78	20.06	0.000	0.48	2.57	0.123	0.10	212.10	0.000	0.91	96.83	0.000	0.82
	Cont	5.75 ± 1.22	6.75 ± 1.29	0.87	0.360	0.04									
Depression	Exp	6.75 ± 2.14	8.83 ± 1.99	11.23	0.003	0.34	0.54	0.470	0.02	136.93	0.000	0.86	18.59	0.000	0.46
	Cont	6.50 ± 1.93	7.42 ± 1.97	3.83	0.063	0.15									
Anger	Exp	8.50 ± 2.28	9.67 ± 2.67	6.10	0.022	0.22	1.06	0.314	0.05	120.81	0.000	0.85	18.27	0.000	0.45
	Cont	8.08 ± 2.43	8.83 ± 2.62	1.32	0.263	0.06									
Fatigue	Exp	5.25 ± 1.76	8.08 ± 2.23	1.32	0.262	0.06	0.38	0.544	0.02	48.89	0.000	0.69	2.31	0.143	0.10
	Cont	4.92 ± 1.88	6.33 ± 1.97	0.53	0.475	0.02									
Confusion	Exp	13.83 ± 1.85	16.50 ± 1.83	11.88	0.002	0.35	1.73	0.202	0.07	225.28	0.000	0.91	25.03	0.000	0.53
	Cont	13.92 ± 1.83	15.17 ± 2.25	3.25	0.085	0.13									
Vigor	Exp	17.33 ± 6.99	25.50 ± 8.02	12.57	0.002	0.36	0.63	0.435	0.03	227.09	0.000	0.91	29.71	0.000	0.58
	Cont	17.42 ± 6.68	20.75 ± 6.27	2.23	0.150	0.09									

TMD, total mood disturbance.

### 2.4 Individual time-based goal setting

The goal-setting procedure was administered by the team coaching staff. The control group received a generic instruction: “perform at your best” for the post-test. The experimental group followed a systematic procedure to determine an individualized, challenging, and quantifiable 100-m freestyle target based on the goal-range setting model (23, 24). This mathematical model establishes a target performance range using each swimmer's five fastest competition times from the preceding three months. For example, given the race performances: 55.14 s (P1), 54.57 s (P2), 54.36 s (P3), 53.94 s (P4), and 53.80 s (P5), the interval is calculated as follows:

A (Mean of top 5 times): (55.14 + 54.57 + 54.36 + 53.94 + 53.80)/5 = 54.36 s.

B (Best performance = lower boundary): 53.80 s.

C (Difference A – B): 54.36–53.80 = 0.56 s.

D (Midpoint value): 53.80–0.56 = 53.24 s.

E (Upper boundary): 53.24–0.56 = 52.68 s.

Thus, the swimmer's target range for the post-test was 52.68–53.24 s. This range, defined as ambitious yet achievable, was individually communicated to each swimmer in the experimental group immediately after the pre-test. Coaches reinforced this objective three times over the eight-week training period to sustain motivation and attentional focus.

### 2.5 100-m freestyle performance measurement

Performance in the 100-m freestyle was assessed during the pre-test and post-test sessions using a standardized protocol. All trials were conducted in a 25-m pool at the same time of day (18:00–20:00) to control for circadian variation. Swimmers completed a standardized 800-m warm-up consisting of aerobic swimming, technical drills, and progressive sprints. Immediately after warming up, each swimmer performed a maximal-effort 100-m freestyle swim from a standard dive start. Performance time was recorded using a Seiko S141 electronic handheld stopwatch (Seiko Holdings Corporation, Tokyo, Japan), a model validated and widely used in swimming research (25). The timekeeper was positioned at the pool finish and remained blind to group assignment.

### 2.6 Psychological and TMD measurement

Psychological state was evaluated using the French version of the Profile of Mood States (POMS), validated for use in sport contexts (26, 27). The instrument measures six mood dimensions across 65 items rated on a 5-point Likert scale (0 = “not at all” to 4 = “extremely”) based on the participant's current state. The subscales assessed were: Tension (anxiety, nervousness; 9 items, 0 to 36), Depression (sadness, low mood; 15 items, 0 to 60), Anger (hostility, irritation; 12 items, 0 to 48), Vigor (energy, vitality; 8 items, 0 to 32), Fatigue (exhaustion, low energy; 7 items, 0 to 28), and Confusion (disorientation, cognitive disturbance; 7 items, 0 to 28). Total Mood Disturbance (TMD) was calculated using the standard formula:

$$\begin{array}{r} \mathrm{Total}\;\mathrm{mood}\;\mathrm{disturbance}=\;[\mathrm{Tension}+\mathrm{Depression}+\mathrm{Anger}\\ +\mathrm{Fatigue}+\mathrm{Confusion}]-\mathrm{Vigor} \end{array}$$

The questionnaire was administered under controlled conditions 30 min before the 100-m performance test during both pre- and post-test sessions. Internal consistency, assessed using Cronbach's alpha, was excellent (*α* = 0.91), confirming high measurement reliability.

### 2.7 Statistical analysis

All statistical analyses were performed using JASP (version 0.95.4, University of Amsterdam, Amsterdam, The Netherlands). Normality was verified using the Shapiro–Wilk test, and homogeneity of variance was assessed using Levene's test. A 2 × 2 mixed-design ANOVA (Time: Pre vs. Post × Group: Experimental vs. Control) was used to analyze the effects of the intervention on performance and psychological outcomes. When significant interaction effects were identified, Holm-Bonferroni-adjusted pairwise comparisons were conducted. Effect sizes were expressed as partial eta-squared (*η*p^2^) and interpreted as small (0.01–0.05), moderate (0.06–0.13), or large (≥ 0.14). Statistical significance was set at *p* < 0.05.

## 3 Results

### 3.1 100-m freestyle performance

In the experimental group, all swimmers improved their 100-m freestyle performance from pre- to post-test. Among them, 9 out of 12 swimmers (75.0%) successfully achieved their individualized time-based performance goal.

**Figure 2:**
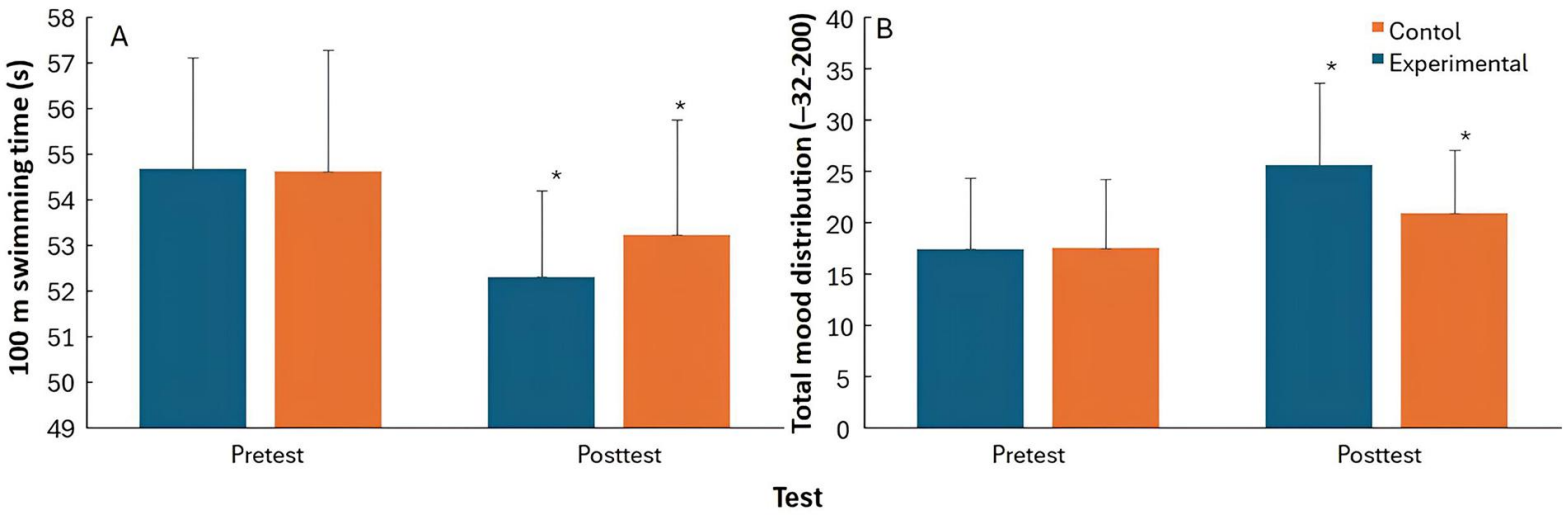
Changes in 100-m freestyle performance time **(A)** and total mood disturbance **(B)** Pre- and post-intervention for the experimental and control groups.

The mixed ANOVA revealed a significant main effect of time on 100-m performance (F = 151.77, *p* < 0.001, *η*p^2^ = 0.87), indicating that swimming speed improved across measurements. A significant Time × Group interaction was also observed (F = 10.04, *p* = 0.004, *η*_p_^2^ = 0.31). Both groups had a significant improvement after the training period. However, the improvement of the experimental group was larger than the control group (4.3 vs. 2.5%, Figure 2A).

### 3.2 Psychological variables (POMS subscales)

Also a significant main effect of time was found for Total Mood Disturbance (TMD) (F = 241.34, *p* < 0.001, *η*p^2^ = 0.92), together with a significant Time × Group interaction (F = 42.63, *p* < 0.001, *η*p^2^ = 0.66) with no significant group effect (F = 0.67, *p* = 0.42, *η*p^2^ = 0.03). Both groups increased their total mood disturbance from pre- to post test, but the increase of the experimental group was significantly higher than of the control group (47.1 vs. 19.1%, Figure 2B).

When investigating the different subscales of the total mood distribution, all subscales demonstrated a significant main effect of time (F ≥ 48.89, *p* < 0.001, *η*p^2^ ≥ 0.31) and a significant Time × Group interaction in all subscales (F ≥ 10.04, *p* < 0.004, *η*p^2^ ≥ 0.82) except for fatigue (F = 2.31, *p* = 0.143, *η*p^2^ = 0.10). No significant group effect was found for any of the subscales (F ≤ 2.57, *p* ≥ 0.123, *η*p^2^ ≤ 0.10).

**Figure 3:**
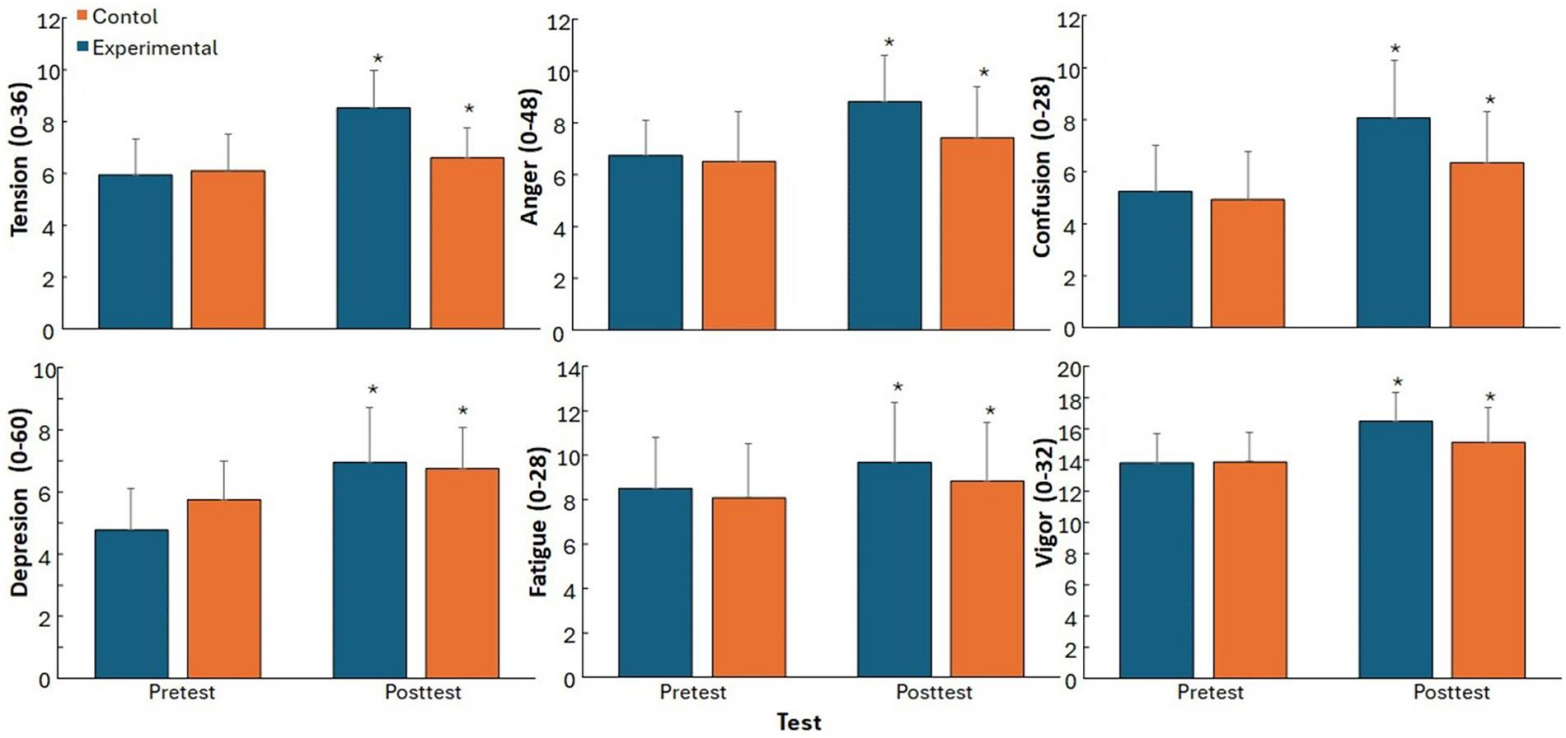
Pre- to post-intervention percentage change in profile of mood states (POMS) subscale scores for the experimental and control groups.

Post hoc comparison revealed from pre- to post testing all subscales increased significantly in both groups, but the increase was significantly larger in the experimental group compared to the control group in each subscale except for fatigue, in which the increase was not significantly different (13.7 vs. 9.3%, Figure 3).

## 4 Discussion

This study demonstrates that setting a time-based performance goal exerts a dual and significant influence. The performance-related hypothesis is supported, as the experimental group showed a significantly greater improvement in 100-m freestyle performance than the control group (4.3 vs. 2.6%). In line with the psychological hypothesis, more pronounced changes in total mood disturbance and in all POMS subscales except fatigue were observed in the experimental group compared with the control group.

The superior improvement observed in the group receiving an individualized time-based goal is consistent with existing literature on goal-setting in sport (28, 29). Locke and Latham (28) report that specific and measurable goals can lead to performance improvements ranging from 3% to 8%, depending on the training load and nature of the motor task. Similarly, a recent meta-analysis by Williamson et al. (29) indicates that the average effect of goal-setting on sport performance corresponds to an improvement of approximately 5% compared with generic instructions such as “do your best.” The present improvement (4.3%) aligns with this expected range and highlights the potential structuring function of personalized goal prescription. At a theoretical level, Goal Setting Theory (15, 28) proposes that precise goals operate through four key cognitive mechanisms: attentional focus, effort mobilization, strategic adjustment, and persistence. In the current study, these mechanisms appear to have enhanced swimmers’ physiological adaptation to the training stimulus. More specifically, the time-based goal likely acted as a motivational catalyst, structuring effort and optimizing behavioral engagement during the training cycle, which explains the differential progression observed between groups.

However, this performance enhancement was accompanied by a marked psychological cost. The experimental group showed a substantial increase in TMD (47%), compared with a more moderate rise in the control group (19%). These findings align with Kovács et al. (30), who observed that demanding goal structures in youth aquatic athletes can increase cognitive anxiety (30%) and emotional tension (25%). Stone et al. (31) similarly demonstrate that competitive goal pressure elevates perceived stress (18%–35%) and negatively alters affective states during tapering phases. Although vigor increased slightly in both groups, negative affective components (tension, anger, anxiety, depression, and confusion) increased disproportionately in the experimental group. This reflects an imbalance between motivational activation and psychological well-being, a pattern described in stress-response frameworks such as the Competitive Stress Model (32). From this perspective, the presence of a fixed time-performance goal may heighten perceived evaluative threat and induce continuous self-comparison, particularly in developing athletes whose self-regulation capacity is still emerging. Thus, although goal-setting supported physical progression, the rise in negative emotional components exceeded the increase in vigor, producing an overall deterioration in mood regulation as reflected by the TMD increase.

These findings suggest that individualized time-based goal-setting may serve as an effective performance-enhancing strategy, yet it must be implemented with psychological monitoring and support. Combining goal-setting with process-oriented goals and autonomy-supportive communication, as proposed in Self-Determination Theory (33), may help preserve intrinsic motivation and emotional stability. Integrating mental preparation techniques such as imagery, motivational self-talk, emotion regulation strategies, and weekly mood profiling may further ensure that performance improvements do not come at the expense of psychological well-being. A progressive introduction of goal difficulty and athlete-co-constructed targets may also reduce emotional overload while maintaining the motivational benefits of goal specification.

This study presents several limitations that should be considered. The sample size was relatively small and consisted exclusively of competitive swimmers, which restricts the generalization of these findings to older or elite populations. A no coping or psychological support strategies were integrated into the intervention, leaving open the possibility that a combined approach might have produced gains in performance without the observed increase in TMD. Future research should examine goal-setting frameworks across age categories, incorporate mental skills training, compare result vs. process-based goals, and explore longer-term adaptations.

## 5 Conclusion and practical recommendations

This study demonstrates that setting a personalized time-based performance goal is an effective strategy to improve swimming performance; however, it is accompanied by notable psychological alterations, particularly a marked increase in mood disturbance among competitive swimmers. While the observed improvement confirms the potential benefit of establishing specific, measurable, and challenging goals, the substantial rise in TMD indicates that this approach should be applied cautiously to avoid emotional overload that may compromise athletes’ well-being. From a practical standpoint, it is recommended that coaches introduce this strategy progressively, prioritize the co-construction of goals, and combine it with mental preparation techniques to support autonomous motivation and emotional self-regulation. Regular monitoring of psychological indicators, particularly mood state profiles, appears necessary to adjust the mental load associated with goal pursuit. Finally, combining this method with process-oriented goals and individualized motivational support may help sustain the performance benefits while protecting long-term psychological balance.

## Data Availability

The original contributions presented in the study are included in the article/Supplementary Material, further inquiries can be directed to the authors.
